# Establishment of a placental lncRNA-mRNA expression network for early-onset preeclampsia

**DOI:** 10.1186/s12884-024-06481-4

**Published:** 2024-04-27

**Authors:** Ya Chen, Ying Zhang, Siyu Xie, Xiangdong Zhou, Lina Zhu, Yunxia Cao

**Affiliations:** Obstetrics and Gynecology department, The first affiliated hospital of Anhui medical university, Hefei, 230000 China

**Keywords:** LncRNA, mRNA gene regulatory networks, Early-onset preeclampsia

## Abstract

**Background:**

This study aimed to establish a placental long non-coding RNA (lncRNA)-mRNA expression network for early-onset preeclampsia (early-onset PE).

**Methods:**

The RNA sequencing data of the GSE14821 dataset were acquired. Several crucial lncRNAs and mRNAs were exerted based on the differential expression analysis of lncRNA and mRNA. By analyzing the differentially expressed lncRNA and mRNA, we constructed a regulatory network to explore the mechanism of the lncRNA in early onset preeclampsia.

**Results:**

A total of 4436 differentially expressed lncRNAs (DElncRNAs) were identified in early-onset PE placenta samples compared with control placenta samples. Pearson correlation analysis revealed significant correlations between 3659 DElncRNAs and 372 DEmRNAs. KEGG analysis showed that the DEmRNAs were enriched in cytokine-cytokine receptor and hypoxia-inducible factor (HIF)-1 pathways. Several well-known early-onset PE-related mRNAs, such as vascular endothelial growth factor A (*VEGFA*) and VEGF receptor 1 (*FLT1*), were involved in the two pathways. Weighted gene co-expression network analysis and cis-regulatory analysis further suggested the involvement of the two pathways and potential DElncRNA-DEmRNA interactions in early-onset PE. Moreover, the upregulation of representative DElncRNAs, such as *RP11-211G3.3* and *RP11-65J21.3*, and DEmRNAs, such as *VEGFA* and *FLT1*, were validated in clinical placenta samples from patients with early-onset PE by quantitative reverse transcription PCR. Importantly, overexpression of *RP11-65J21.3* significantly promoted the proliferation of HTR-8 trophoblast cells at 72 h after transfection.

**Conclusions:**

In conclusion, we identified placental DElncRNAs of early-onset PE and established a DElncRNA-DEmRNA network that was closely related to the cytokine-cytokine receptor and HIF-1 pathways. Our results provide potential diagnostic markers and therapeutic targets for early-onset PE management.

**Supplementary Information:**

The online version contains supplementary material available at 10.1186/s12884-024-06481-4.

## Background

Preeclampsia is a gestational disorder characterized by hypertension and proteinuria after 20 weeks of gestation, affecting approximately 5–7% of all pregnancies and representing the leading cause of maternal and perinatal mortality worldwide [1, 2]. Despite research endeavors, the etiology of preeclampsia remains elusive. Preeclampsia is classified into early-onset preeclampsia (early-onset PE) and late-onset preclampsia (late-onset PE) according to the gestational age of onset (< 34 weeks and ≥ 34 weeks, respectively). Early-onset PE has more adverse clinical outcomes than late-onset PE and is associated with severe maternal and perinatal complications [3, 4]. Early diagnosis and timely management are essential to lower morbidity and mortality associated with early-onset PE. The placenta is a major damaged organ in preeclampsia and plays a central role in the pathogenesis of preeclampsia [1]. The only cure for preeclampsia is delivery of the fetus and placenta [5]. Thus, analysis of placental genomic alterations related to early-onset PE may help unveil the cause of early-onset PE and identify potential diagnostic biomarkers for early-onset PE.

Long non-coding RNAs (lncRNAs) are a class of non-coding RNA molecules containing more than 200 nucleotides, serving as architectural RNAs, microRNA (miRNA) sponges, or regulators of cellular functions [6]. LncRNAs can bind miRNAs and prevent the regulatory effect of miRNAs on mRNAs, acting as competitive endogenous RNAs or miRNA sponges [7]. Dysregulation of lncRNAs is associated with various human diseases, including preeclampsia [8]. Studies have reported abnormal lncRNA expression profiles of human umbilical vein endothelial cells, plasma exosomes, and placenta from patients with early-onset PE. Bioinformatics analysis has shown that the dysregulated lncRNAs contribute to the pathogenesis of early-onset PE through multiple pathways, including p53, JAK/STAT, PI3K-Akt, and cell adhesion molecules signaling pathways [9–11]. However, the interaction between differentially expressed lncRNAs (DElncRNAs) and DEmRNAs in the early-onset PE placenta remains largely unknown.

In this study, we identified placental DElncRNAs in early-onset PE patients from the GSE148241 dataset, constructed a DElncRNA-DEmRNA expression network, and identified the signaling pathways associated with the network. The dysregulation of the DElncRNA-DEmRNA network was validated in clinical samples from patients with early-onset PE. Our results may provide new insights into the pathogenesis of early-onset PE and potential diagnostic markers and therapeutic targets for early management of early-onset PE.

## Methods

### Patients and sample collection

Placenta samples were collected from 5 early-onset PE patients and 5 normal controls in the department of obstetrics, The First Affiliated Hospital of Anhui Medical University (Anhui, China) between February 2021 and June 2021. Based on the guidelines of the American College of Obstetricians and Gynecologists [12], the diagnostic criteria of preeclampsia were as follows: hypertension (systolic blood pressure ≥ 140 mmHg or diastolic blood pressure ≥ 90 mmHg on two occasions at least 4 h apart after 20 weeks of gestation) accompanied by proteinuria or hypertension accompanied by clinical symptoms such as renal insufficiency, elevated liver enzymes, headache, and epigastric discomfort feelings. Early-onset PE was defined as preeclampsia occurring before 34 weeks of gestation. Patients with diabetes mellitus, multiple pregnancies, intrahepatic cholestasis of pregnancy, or other systematic complications and chronic diseases were excluded. The normal controls were women who received cesarean section before 34 weeks of gestation due to indications other than preeclampsia. The normal controls showed no clinical signs and symptoms of preeclampsia. The tissue samples were collected from the central part of the placenta immediately after the cesarean section. After washing with phosphate-buffered saline buffer, samples were stored at -80 ℃ until use. This study was approved by the Ethics Committee of The First Affiliated Hospital of Anhui Medical University (approval #: 5,101,028). All participants provided informed consent.

### RNA sequencing (RNA-seq) data processing

The datasets generated and analysed during the current study are available in the of the Gene Expression Omnibus database (https://www.ncbi.nlm.nih.gov/geo/query/acc.cgi?acc=GSE148241), containing RNA expression profiles of the placenta tissue samples from 9 patients with early-onset PE and 32 normal controls. The sequencing data were obtained from the Sequence Read Archive (SRA) and converted to fastq format using NCBI SRA Tool fastq-dump. Low-quality bases were removed from the raw reads using a FASTX-Toolkit (v.0.0.13; http://hannonlab.cshl.edu/fastx_toolkit/) to generate clean reads, followed by quality control procedures including sequencing quality distribution analysis, distribution analysis of clean tag length, GC content measurement, and analysis of PCR duplication level using the FastQC (http://www.bioinformatics.babraham.ac.uk/projects/fastqc). The high-quality clean reads were subjected to further analysis.

### Identification of DElncRNAs and DEmRNAs

**Fig. 1:**
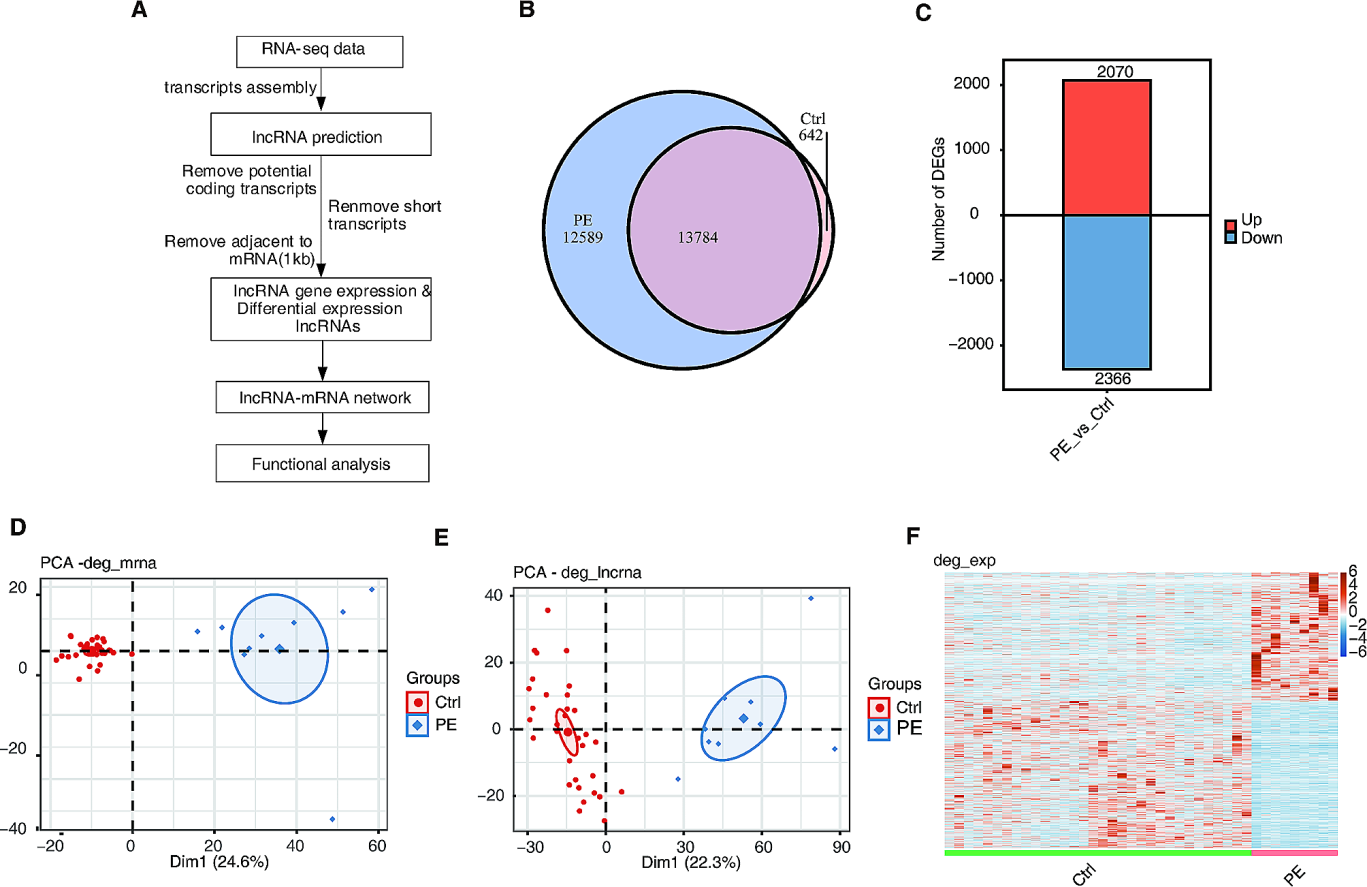
Identification of early-onset preeclampsia (early-onset PE)-related differentially expressed lncRNAs (DElncRNAs) in the GSE14821 dataset. (**A**) RNA sequencing data of the GSE148241 dataset were acquired. The workflow diagram was shown. (**B**) Venn diagram of lncRNAs expressed in early-onset PE and control placenta tissue samples was shown. LncRNAs with reads per kilobase of exon per million fragments mapped (RPKM) ≥ 0.2 in at least two samples were included. (**C**) The number of upregulated and downregulated DElncRNAs. **D**, **E**. Principal component analysis (PCA) of DElncRNAs and DEmRNAs. The samples were grouped by disease state. The ellipse indicates the 95% confidence regions of each group. **F**. Heatmap of DElncRNA expression in placenta samples of early-onset PE group versus control group. PE, preeclampsia; Ctrl, control

Clean reads were aligned to the human GRch38 genome using TopHat2 [13]. Uniquely mapped reads were used to calculate read numbers and reads per kilobase of exon per million fragments mapped for each gene. DEmRNAs were identified using edgeR [14]. mRNAs with the fold change ≥ 2 or ≤ 0.5 and false discovery rate (FDR) ≤ 0.05 were considered DEmRNAs. DElncRNAs were identified following a workflow (Fig. 1A) constructed using the Cufflinks software as previously described [15, 16].

### Weighted gene co-expression network analysis (WGCNA)

DElncRNA and DEmRNA co-expression was analyzed using WGCNA as previously described [17]. Eigengenes of each clustering module were used as the representative expression pattern of genes in each module. Pearson’s correlation coefficients were used to examine the correlations between lncRNAs and mRNAs.

### Gene ontology (GO) and kyoto encyclopedia of genes and genomes (KEGG) analyses

GO and KEGG pathways analyses were performed to characterize the functions of DEmRNAs using KOBAS 2.0 [18]. The enrichment of each term was determined using the hypergeometric test and Benjamini-Hochberg FDR controlling procedure. Functional enrichment analysis of the selected gene sets was conducted using Reactome (http://reactome.org).

### Identification of miRNAs targetd by five DElncRNAs

MiRanda (version v3.3a) database was used to screen miRNAs targeted by 5 differentially expressed lncRNAs. Then we identified putative mRNA targets for miRNAs in the miRDB (version 6.0) and TargetScan (version 8.0) databases. Combining miRNAs target gene and DEGs, we used the Cytoscape software to explore the lncRNA-miRNA-mRNA network with the interactions of results. This gene dataset was subjected to pathway and process enrichment analysis with the Metascape bioinformatics online tool (www.metascape.org) that uses several ontology sources: KEGG Pathway, GO Biological Processes, Reactome Gene Sets, Canonical Pathways, CORUM, and WikiPathways.

### Cell culture and transfection

Human extravillous trophoblast cell line HTR-8 was acquired from procell (Wuhan, Hubei, China) and maintained in RPMI-1640 supplemented with 10% fetal bovine serum, 100 g/mL streptomycin, and 100 U/mL penicillin in an atmosphere of 5% CO_2_ at 37 °C. pcDNA3.1 vectors expressing lncRNA* RP11-65J21.3* were purchased from Youbio Biotech (Changsha, Hunan, China). HTR-8 cells were transfected with the vectors using Lipofectamine 2000 (Invitrogen, Carlsbad, CA, USA) following the manufacturer’s instructions.

### Cell counting kit-8 (CCK-8) assay

CCK-8 assay (#HY-K0301; MCE, Monmouth Junction, NJ, USA) was performed to determine the proliferation of HTR-8 cells according to the manufacturer’s protocols. Cells were harvested at 24 h, 48 h, or 72 h after transfection. The absorbance was measured at 450 nm using a microplate reader (Thermo Fisher Scientific, Waltham, MA, USA). The experiment was performed in triplicate.

### Quantitative reverse transcription PCR (qRT-PCR)

qRT-PCR was performed to verify the differential expression of DEmRNAs and DElncRNAs in placenta samples from early-onset PE patients and normal controls. The primer sequences were summarized in Table 1. qRT-PCR was conducted using the Yeason SYBR mix (Takara, Shiga, Japan). GAPDH was used as an internal control. The relative expression of each gene was expressed as fold changes using the 2^∆∆Ct^ method.

**Table 1:** Primer sequences

Gene	Forward primer (5’–3’)	Reverse primer (5’–3’)
GAPDH	GGTCGGAGTCAACGGATTTG	GGAAGATGGTGATGGGATTTC
FLT1	TCTTGGTCAGGCTGGTCTTG	CCATCGTCATCGTCATCATCAC
VEGFA	CCATCGTCATCGTCATCATCAC	AGGAAGGTCAACCACTCACA
LEP	GCAGTGAGTTACAGCGAGAG	CTGATTAGGTGGTTGTGAGGAT
SH3BP5-AS1	GGCAGATCCTCCACAGATGT	CCCTGAAGAACCTGGAGATGA
RP11-211G3.3	TTATGGGCTCTAAACTGCTCAC	ATGCCAGTGATGTTCTTCTCAA
RP5-1112D6.4	CGTGCTGTTCTTGTGATAGTGA	ATCATGGCGGAAGGCAAGG
RP11-488P3.1	TGTGTTTCCAAGCGGTGTTT	TCTAAGCCAGCGAGACATCC
RP11-65J21.3	GCTTGGCTTGGAATCCTCTC	GGACTCATACTGGGCTCATTTC

### Statistical analysis

The qRT-PCR data were expressed as the mean ± standard deviation. Statistical analysis was performed using Prism (GraphPad Software, Inc., La Jolla, CA, USA). Comparisons between two groups were conducted using two-way ANOVA and Student’s t-test. The clustering of placenta samples was demonstrated by principal component analysis (PCA) using R package factoextra (https://cloud.r-project.org/package=factoextra). Heatmap was generated using the heatmap package (https://cran.rproject.org/web/packages/pheatmap/index.html) in R. A *P* value less than 0.05 was considered statistically different.

## Results

### Identification of early-onset PE-related DElncRNAs

A total of 4671 known lncRNAs and 22,344 novel lncRNAs were identified in all placenta samples from the GSE14821 dataset (Fig. S1A). The characteristics of lncRNAs and mRNAs are shown in Fig. S1B–E, including the distribution of exon count and length of RNAs as well as sample clustering according to lncRNA and mRNA expression. Specifically, we acquired 26,373 lncRNAs in early-onset PE placenta samples and 14,426 lncRNAs in control placenta samples (Fig. 1B). Of these lncRNAs, we identified 4436 DElncRNAs in early-onset PE samples compared with those in control samples, including 2070 upregulated DElncRNAs and 2366 downregulated DElncRNAs (Fig. 1C). The results of PCA showed that early-onset PE and control samples were well separated into two clusters by the expressions of DEmRNAs and DElncRNAs (Fig. 1D and E). The heatmap of DElncRNA expression showed that early-onset PE and normal control placenta samples exhibited different lncRNA expression patterns (Fig. 1F). These data suggest that the lncRNA expression profile may distinguish early-onset PE placental samples from control placenta samples.

### Correlation analysis of DElncRNAs and DEmRNAs

**Fig. 2:**
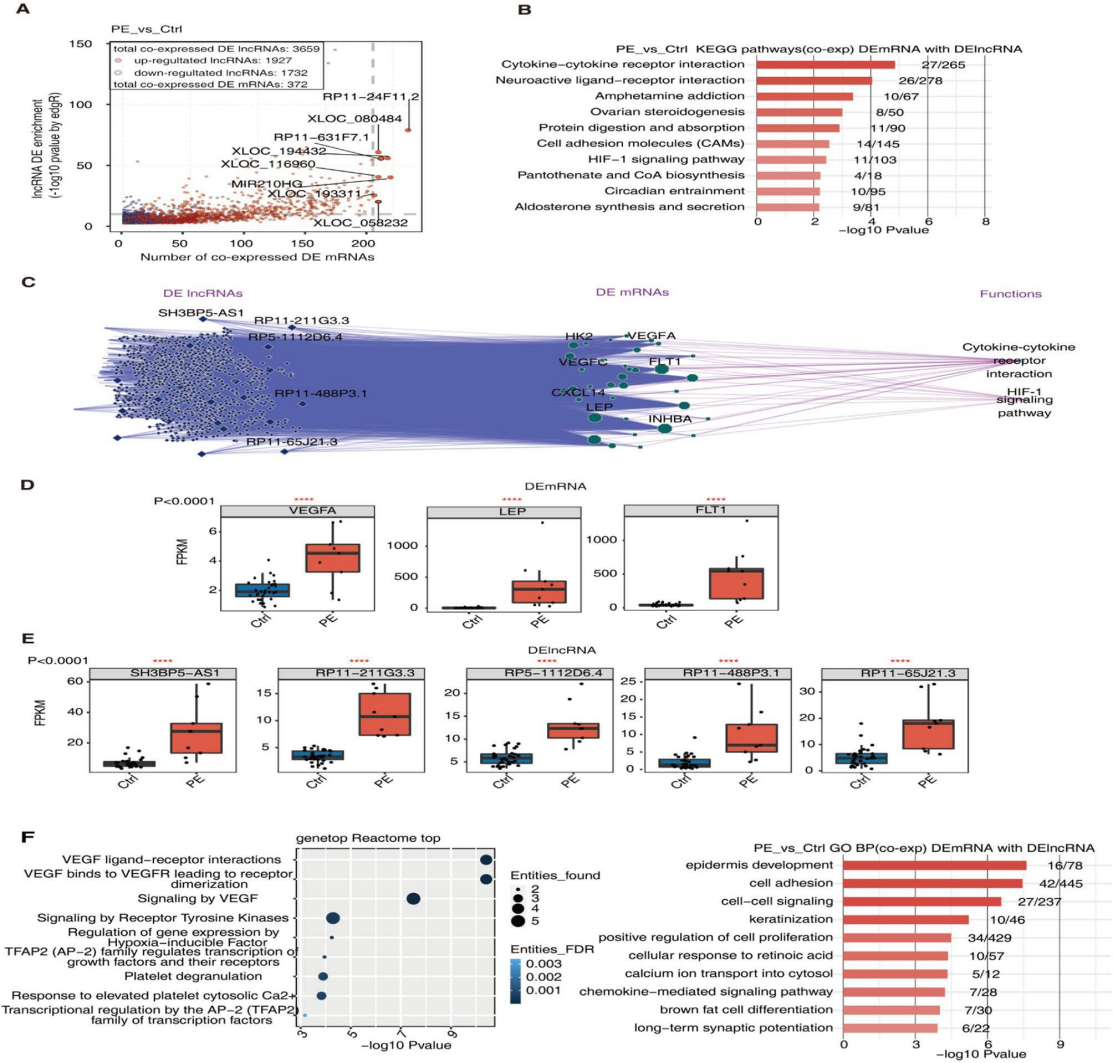
Correlation between DElncRNAs and DEmRNAs. A. A scatter plot of Pearson correlation coefficients of DElncRNAs with DEmRNAs. Red dots represent upregulated lncRNAs. Blue dots represent downregulated lncRNAs. A DEmRNA-DElncRNA pair with a *P* value < 0.01 and Pearson coefficient > 0.6 was considered significantly correlated. **B**. The top 10 enriched KEGG pathways of DEmRNAs correlated with DElncRNAs. **C**. The DElncRNA-DEmRNA-pathway network. **D**, **E.** Boxplots of RPKMs of representative correlated DEmRNAs (**D**) and DElncRNAs (**E**). **F.** The top 10 enriched Reactome pathways (left) and GO biological process (right) of DEmRNAs correlated with DElncRNA

To identify potential DEmRNA targets of DElncRNAs, we performed Pearson correlation analysis. We observed significant correlations between 3659 DElncRNAs and 372 DEmRNAs. Of the 3659 DElncRNAs, 1927 were upregulated whereas 1732 were downregulated (Fig. 2A). For the first time, we found some novel early-onset PE-related DEmRNAs, such as *FAM47E-STBD1* and *MTNR1B*, and novel early-onset PE-related DElncRNAs, such as *RP11-631F7.1*, *RP11-65J21.3*, *XLOC_058232*, *XLOC_193299*, *RP11-24F11.2*, and *XLOC_080484*. KEGG analysis showed that the DEmRNAs correlated with DElncRNAs were enriched in cytokine-cytokine receptor interaction and hypoxia-inducible factor (HIF-1) signaling pathways (Fig. 2B) that are involved in the pathogenesis of preeclampsia [19, 20]. Further, we found early-onset PE-related DEmRNAs in the two pathways, including hexokinase 2 (*HK2*), vascular endothelial growth factor A (*VEGFA*), *VEGFC*, VEGF receptor 1 (*FLT1*), leptin (*LEP*), chemokine ligand 14, and inhibin subunit beta A (*INHBA*) (Fig. 2C) [21–27]. These DEmRNAs were correlated with DElncRNAs *SH3BP5-AS1*, *RP11-211G3.3*, *RP5-1112D6.4*, *RP11-488P3.1*, and *RP11-65J21.3*. Moreover, *VEGFA*, *LEP*, *FLT1*, *SH3BP5-AS1*, *RP11-211G3.3*, *RP5-1112D6.4*, *RP11-488P3.1*, and *RP11-65J21.3* expressions were remarkably upregulated in early-onset PE group compared with those in control group (Fig. 2D and E), suggesting that these DEmRNAs and DElncRNAs may serve as potential diagnostic markers for early-onset PE. Figure 2F shows the top 10 biological processes and top 10 signaling pathways enriched in DEmRNAs of early-onset PE.

### Co-expression analysis of all lncRNAs and mRNAs expressed in early-onset PE and control placenta samples

**Fig. 3:**
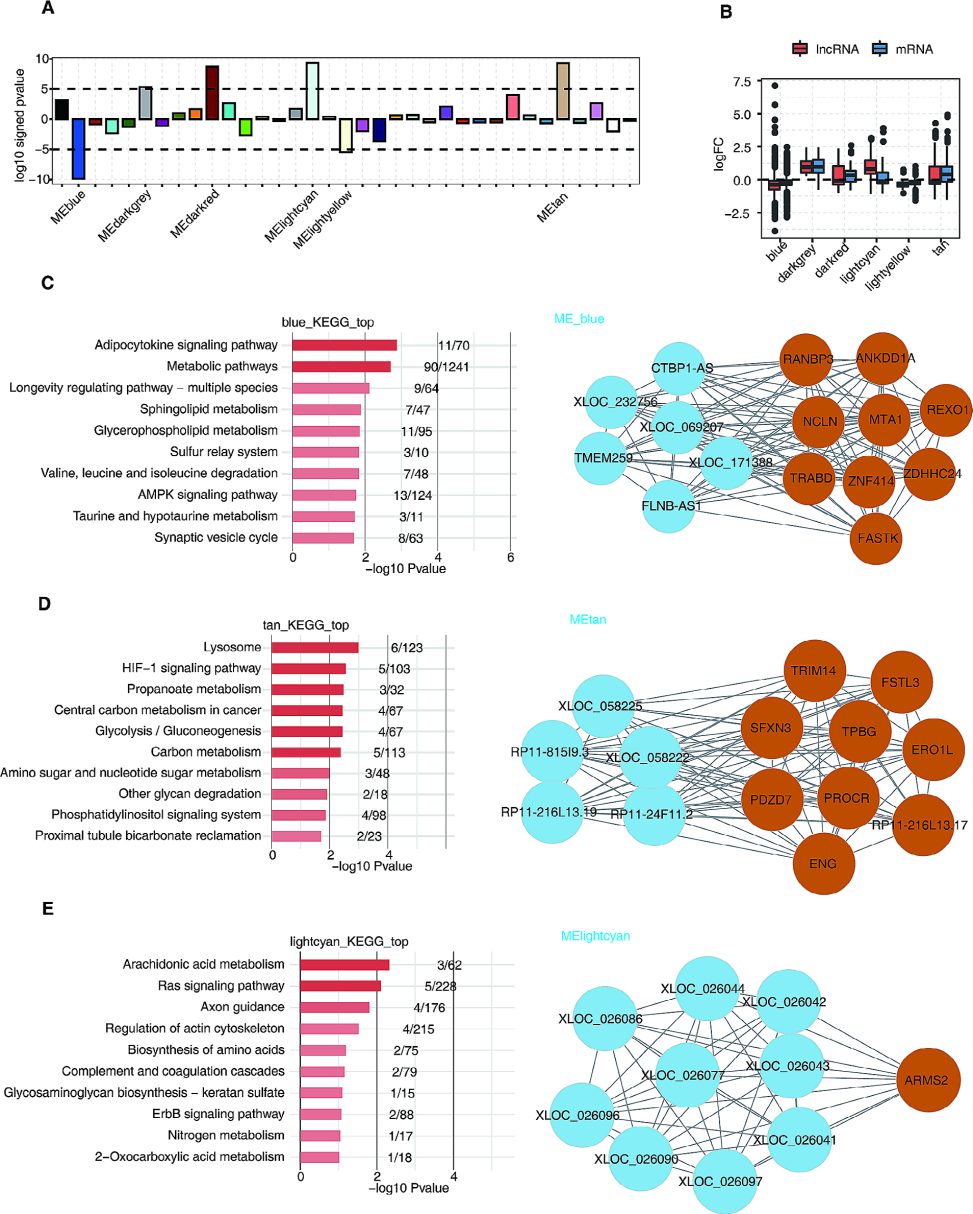
Weighted gene co-expression network analysis (WGCNA) of all lncRNAs and mRNAs expressed in early-onset PE and control groups. **A**. WGCNA assigned all lncRNAs and mRNAs expressed in early-onset PE and control groups into different co-expression modules. Positive and negative values indicate modules with increased and decreased expressions respectively, in early-onset PE group compared with those in control group. Dashed lines signify associated modules. **B**. A boxplot of expression levels (log fold change) of mRNAs and lncRNAs of each module. **C**–**E**. The network of hub mRNAs and lncRNAs (right) and the KEGG pathways enrichment assay (left) of blue (**C**), tan (**D**), and light cyan (**E**) modules. Brown circles indicate hub mRNAs. Blue circles indicate hub lncRNAs

To identify potential lncRNA-mRNA pairs related to early-onset PE, we performed WGCNA to examine lncRNA and mRNA co-expression. As shown in Fig. 3A, 6 co-expression modules were generated and assigned different colors, including blue, dark grey, dark red, light cyan, light yellow, and tan modules containing 1829, 87, 116, 184, 169, and 295 genes, respectively. Figure 3B displays the expression levels of lncRNAs and mRNAs in each module. We noticed that the lncRNAs and mRNAs in blue and light yellow modules tended to be downregulated whereas those in dark grey, dark red, light cyan, and tan modules were generally upregulated in early-onset PE (Fig. S2A–C). Then, we conducted KEGG enrichment analysis on the 6 modules. As shown in Fig. 3C–E and Fig. S2D–F, the gene modules were enriched in HIF-1 signaling and inflammation-related pathways (left). We also found potential lncRNA-mRNA pairs (right) that may play important regulatory roles in the pathogenesis of early-onset PE.

### Identification of cis-target mRNAs of DElncRNAs

**Fig. 4:**
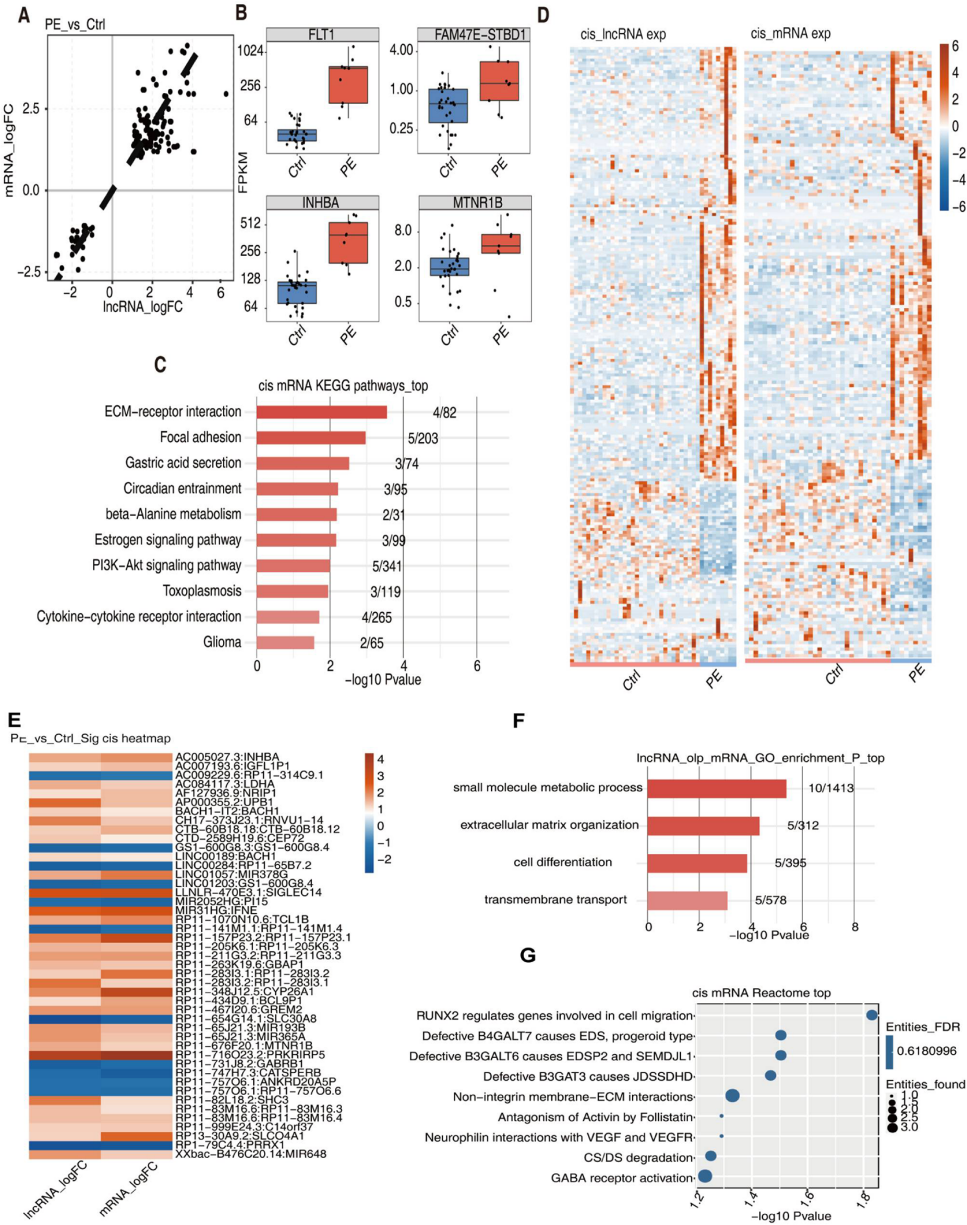
Identification of cis-target mRNAs of DElncRNAs. **A**. A scatter plot of expressions (logFC) of DElncRNAs and cis-target mRNAs. **B**. Box plots of expressions (RPKM) of representative cis-target mRNAs in early-onset PE and control placenta samples. **C**. The top 10 enriched KEGG pathways of cis-target mRNAs. **D**. Heatmap of the expressions of DElncRNAs and cis-target mRNAs. **E**. Heatmap of the expressions of DElncRNA-cis-mRNA pairs. **F**. The top 10 GO biological processes of cis-target mRNAs. **G**. The Top 10 enriched reactome pathways of cis-target mRNAs. PE, preeclampsia; Ctrl, control

We further performed cis-acting analysis on 4436 DElncRNAs and identified 184 cis-target mRNAs (Fig. 4A), including *FLT1*, *FAM47E-STBD1*, *INHABA*, and *MTNR1B* that were substantially upregulated in early-onset PE group compared with those in control group (Fig. 4B). KEGG analysis showed that the cis-target mRNAs were significantly associated with the cytokine-cytokine receptor, ECM-receptor interaction, and PI3K-Akt signaling pathways (Fig. 4C). The heatmap showed that the DElncRNAs and cis-target mRNAs exhibited similar expression patterns between early-onset PE and control groups (Fig. 4D), suggesting a positive regulatory relationship between DElncRNAs and cis-target mRNAs. The expression patterns of representative lncRNA-mRNA pairs are shown in Fig. 4E. The cis-target mRNAs were mainly enriched in biological processes of small molecule metabolic process, extracellular matrix organization, and cell differentiation (Fig. 4F) as well as the reactomes involving *RUNX2*, *B4GALT7*, and *B3GALT6*(Fig. 4G).

### Identification of the miRNAs targeted by identified DElncRNAs

**Fig. 5:**
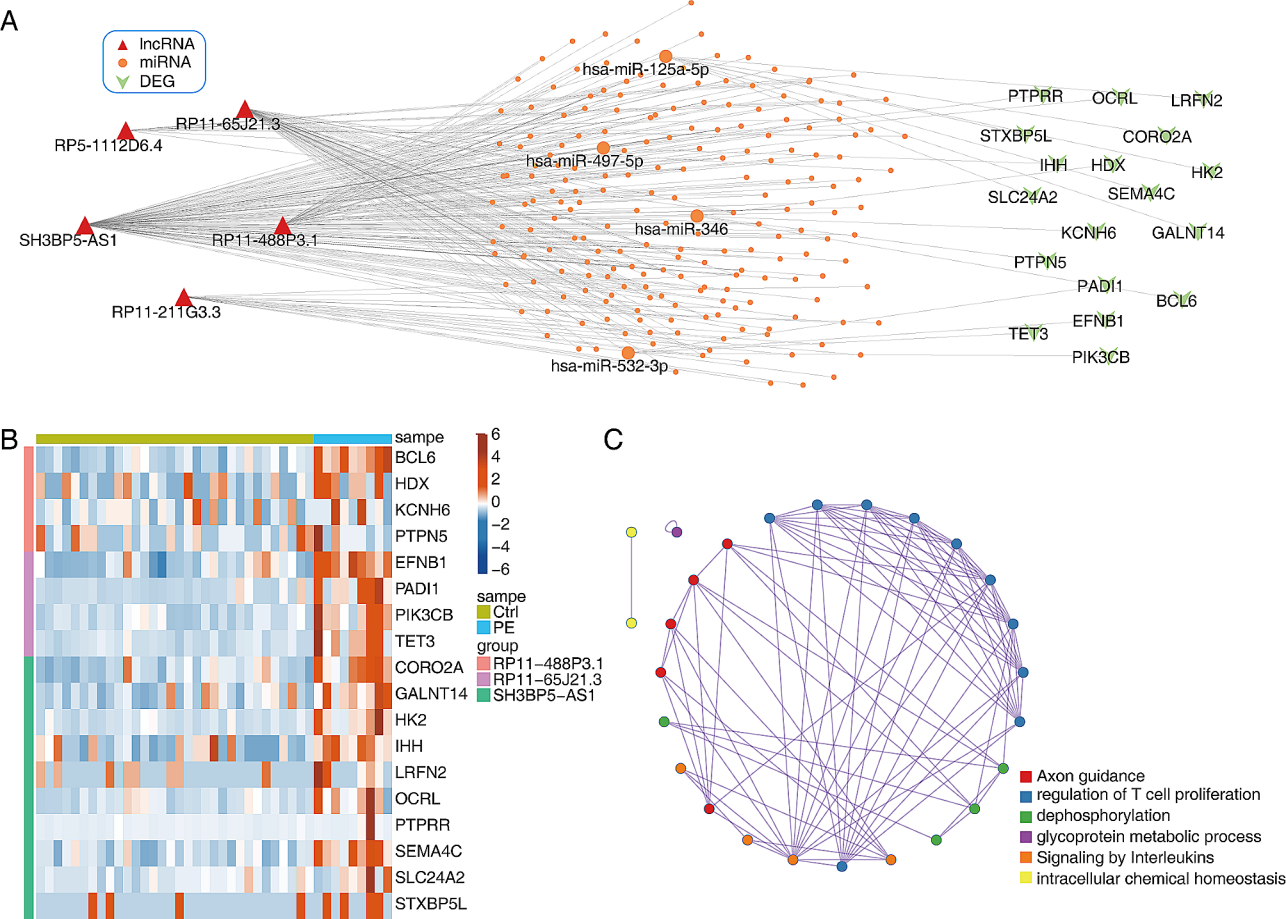
Identification of miRNAs targetd by five DElncRNAs. A. Network of DElncRNAs-miRNAs-DEGs for PE vs. Ctrl samples. **B**, **C**. Hierarchical clustering heat map showing expression levels of all DEGs. **C**. Metascape enrichment network visualization showing the enriched terms of DEGs. Cluster annotations are shown in color cod

Network of five DElncRNAs-miRNAs-DEGs for early-onset PE compared with control samples was shown in Fig. 5A. Four key miRNAs were identified as putative targets for DElnRNAs, including *has-miR-125a-5p*, *has-miR-497-5p*, *has-miR-346* and *has-miR-532-3p*. Based on the interact results of the lncRNA-miRNA-mRNA network, DEGs between early-onset PE and control samples were furtherly enriched in several pathways, such as regulation of T cell proliferation and glycoprotein metabolic process (Fig. 5BC).

### Validation of abnormal expressions of representative DElncRNAs and DEmRNAs in clinical specimens

**Fig. 6:**
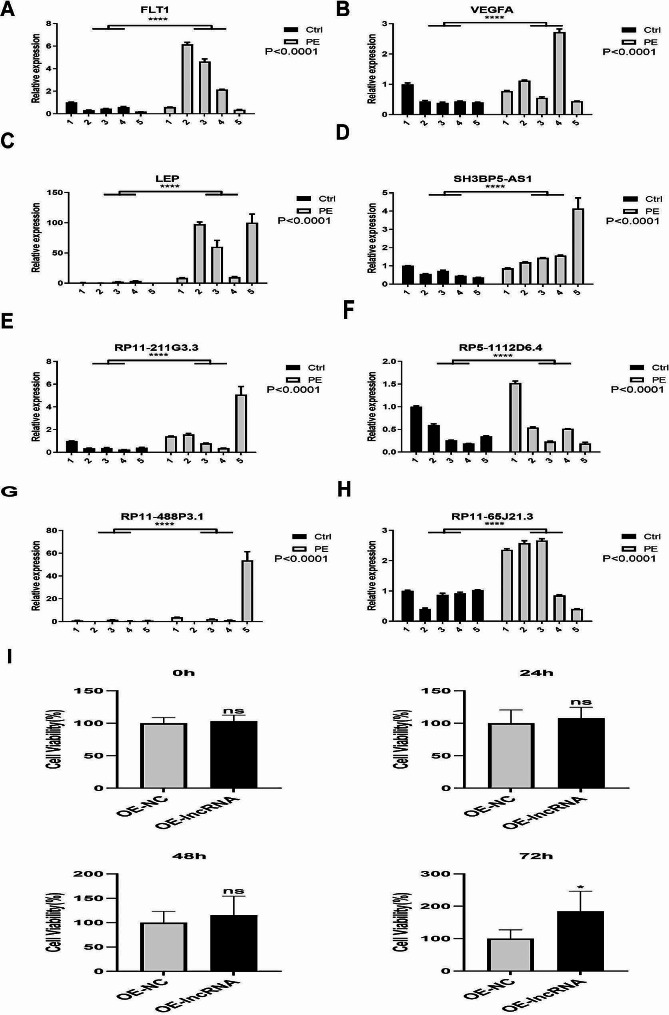
Validation of abnormal expressions of representative DElncRNAs and DEmRNAs in clinical specimens. qRT-PCR was performed to determine DEmRNAs (*FLT1*, *VEGFA*, and* LEP*) (**A, B,C**) and DElncRNAs (*SH3BP5-AS1*, *RP11-211G3.3*, *RP5-1112D6.4*, *RP11-488P3.1* and* RP11-65J21.3*) (**D, E,F, G,H**) in placenta samples of patients with early-onset PE and normal controls (*n* = 5/group). x-axis: the sample numbers. **I**. HTR-8 cells were transfected with vectors expressing lncRNA* RP11-65J21.3*. CCK-8 assay was performed at 0, 24, 48, and 72 h after transfection. Data are expressed as the mean ± standard deviation. *P < 0.05, *n* = 3

To assess the clinical significance of the RNA-seq results, we conducted qRT-PCR to determine the expression of representative DEmRNAs (*FLT1*, *VEGFA*, and *LEP*) and DElncRNAs (*SH3BP5-AS1*, *RP11-211G3.3*, *RP5-1112D6.4*, *RP11-488P3.1*, and *RP11-65J21.3*) in clinical specimens. The results showed that all the representative DElncRNAs and DEmRNAs were significantly upregulated in early-onset PE placentae compared with those in control placentae (Fig. 6A–H), consistent with the RNA-seq data. Since *RP11-65J21.*3 was upregulated in more patients than other representatives, we overexpressed *RP11-65J21.3* in HTR-8 cells to examine its effect on cell proliferation. CCK-8 assay showed that overexpression of *RP11-65J21.3* significantly promoted HTR-8 cell proliferation at 72 h after transfection (Fig. 6I). These data suggest that DElncRNAs are involved in the pathogenesis of early-onset PE possibly by regulating trophoblast cell proliferation.

## Discussion

In this study, we identified 4436 DElncRNAs in the placenta samples of early-onset PE. Of these DElncRNAs, 3659 were correlated with 372 DEmRNAs. WGCNA revealed potential DElncRNA-DEmRNA pairs related to early-onset PE. Cis-acting analysis identified 184 cis-target mRNAs of DElncRNAs, and the cis-target mRNAs were associated with early-onset PE-related signaling pathways, such as cytokine cytokine-cytokine receptor and HIF-1 pathways. The abnormal expression of representative DElncRNAs and DEmRNAs were further validated in clinical placenta samples of early-onset PE patients by qRT-PCR. Intriguingly, overexpression of *RP11-65J21.3* significantly promoted cell proliferation of HTR-8 cells. Taken together, our study suggests that dysregulation of lncRNAs plays an important role in the pathogenesis of early-onset PE possibly by regulating mRNA expression and trophoblast cell proliferation.

In the present study, we identified several novel early-onset PE-related lncRNAs (*RP11-631F7.1*, *RP11-65J21.3*, *XLOC_058232*, *XLOC_193299*, *RP11-24F11.2*, and *XLOC_080484*) and novel early-onset PE-related mRNAs (*FAM47E-STBD1* and *MTNR1B*). These molecules may serve as potential diagnostic markers and therapeutic targets for early-onset PE. The DEmRNAs correlated with the DElncRNAs were enriched in cytokine-cytokine receptor and HIF-1 pathways that are highly involved in the pathogenesis of early-onset PE, as previously described [28, 29]. The most common pathologic change of early-onset PE is the insufficient invasion of trophoblast cells, leading to inadequate uterine artery remolding by producing excess cytokines and placental debris [25]. Systemic inflammatory response and extensive endothelial damages are responsible for the development of early-onset PE. Compared with late-onset PE, early-onset PE is associated with more pronounced inflammation and aberrant angiogenesis [30]. At the miRNA level, we identified four miRNAs as putative targets for five representative DElnRNAs, including *has-miR-125a-5p*, *has-miR-497-5p*, *has-miR-346* and *has-miR-532-3p*. Researchers identified *has-miR-125a-5p* and *has-miR-532-3p* as inhibition of angiogenesis in other diseases [31, 32]. These two miRNAs could repress the growth, migration, and invasion of vascular smooth muscle cell and ovarian cancer cell respectively. Besides, *has-miR-346* was proved to not only suppress *VEGF* expression, but also inhibit trophoblast invasion and migration in the HTR-8/SV neo cell lines [33]. Additionally, previous study confirmed that *has-miR-497-5p* could attenuate cell growth, migration and invasion through TGF-β signaling pathway [34]. Finally, identified DElncRNAs work as the etiology of early-onset PE through these downstream miRNAs.

It has been reported that TGF-β/Smad signaling is hyperactivated in decidua-embedded extravillous trophoblasts in human early-onset PE placenta samples [35]. TGF-β1 inhibits trophoblast cell migration and invasion, contributing to the development of early-onset PE [36, 37]. In addition, increased soluble FLT1 (sFLT1) is responsible for vascular remodeling disorder in patients with early-onset PE by suppressing VEGF and placenta growth factor signaling [38]. The increased maternal TNF-α may upregulate the expression and release of placental fractalkines, which in turn enhances systemic inflammatory response in early-onset PE [39]. Moreover, the cytokine macrophage migration inhibitory factor not only promotes the production and expression of proinflammatory mediators but also enhances angiogenic biological activities [40], linking the two major etiologic pathways of early-onset PE. The proinflammatory cytokines, angiogenic factors, and the renin-angiotensin system may trigger maternal inflammatory response and vascular dysfunction [41]. We thus speculate that lncRNA dysregulation contributes to the occurrence and development of early-onset PE through inflammation-related pathways.

HIF-1 is a transcription factor that plays a key role in the cellular response to hypoxia [42]. Hypoxia is a predisposing factor of early-onset PE [43]. Studies have shown that patients with preeclampsia are characterized by elevated sFLT1, soluble endoglin, and endothelin-1 levels that are induced by elevated placental HIF-1 levels [44–46]. Additionally, hypoxia-independent stimulators of HIF-1α in the placenta also promote the progression of preeclampsia, such as the pathogenic autoantibody and inflammatory cytokine tumor necrosis factor superfamily member 14 [47]. Although it remains controversial whether early-onset PE patients have increased serum or placental HIF-1α levels compared with late-onset PE patients [48, 49], hypoxia or hypoxia-independent factor-induced HIF-1 upregulation plays an undoubtedly important role in the pathogenesis of early-onset PE.

Our study also identified potential lncRNA-mRNA pairs through cis-regulatory analysis, involving important mRNAs related to early-onset PE, such as *FLT1*, *INHBA*, *FAM47E-STBD1*, and *MTNR1B*. Of these mRNAs, *FLT1* and *INHBA* are associated with cytokine-cytokine receptor pathway and are highly expressed in preeclampsia [23, 50] whereas the roles of *FAM47E-STBD1* and *MTNR1B* in preeclampsia remain unknown. This finding may provide new insights into the pathogenesis of early-onset PE and future research directions.

## Conclusions

We established an early-onset PE-related placental lncRNA-mRNA network, providing potential diagnostic markers and therapeutic targets for early-onset PE management. However, a large cohort is required to validate the results of this study. Future studies are needed to identify stable and representative indicators in lncRNA-mRNA network and to develop a quick-and-safe method for the detection of placental expression of lncRNAs and mRNAs.

### Electronic supplementary material

Below is the link to the electronic supplementary material.

Supplementary Material 1

## Data Availability

The data presented in this study are available in article or supplementary material. The RNA-seq raw data generated and analysed for this study have been deposited in the of the Gene Expression Omnibus database (https://www.ncbi.nlm.nih.gov/geo/query/acc.cgi?acc=GSE148241).

## Abbreviations

early-onset PE: early-onset preeclampsia
late-onset PE: late-onset preeclampsia
PCA: principal component analysis
sFLT1: soluble VEGF receptor 1
