# Supplementary material for: Establishment of a placental lncRNA-mRNA expression network for early-onset preeclampsia
Source: BMC Pregnancy Childbirth. 2024 Apr 27;24:329. doi: 10.1186/s12884-024-06481-4 (PMC11055331; doi:10.1186/s12884-024-06481-4)
Supplement: Supplementary file 1 — Supplementary Material 1 [file 12884_2024_6481_MOESM1_ESM.docx]

**Supplementary Figures**

**
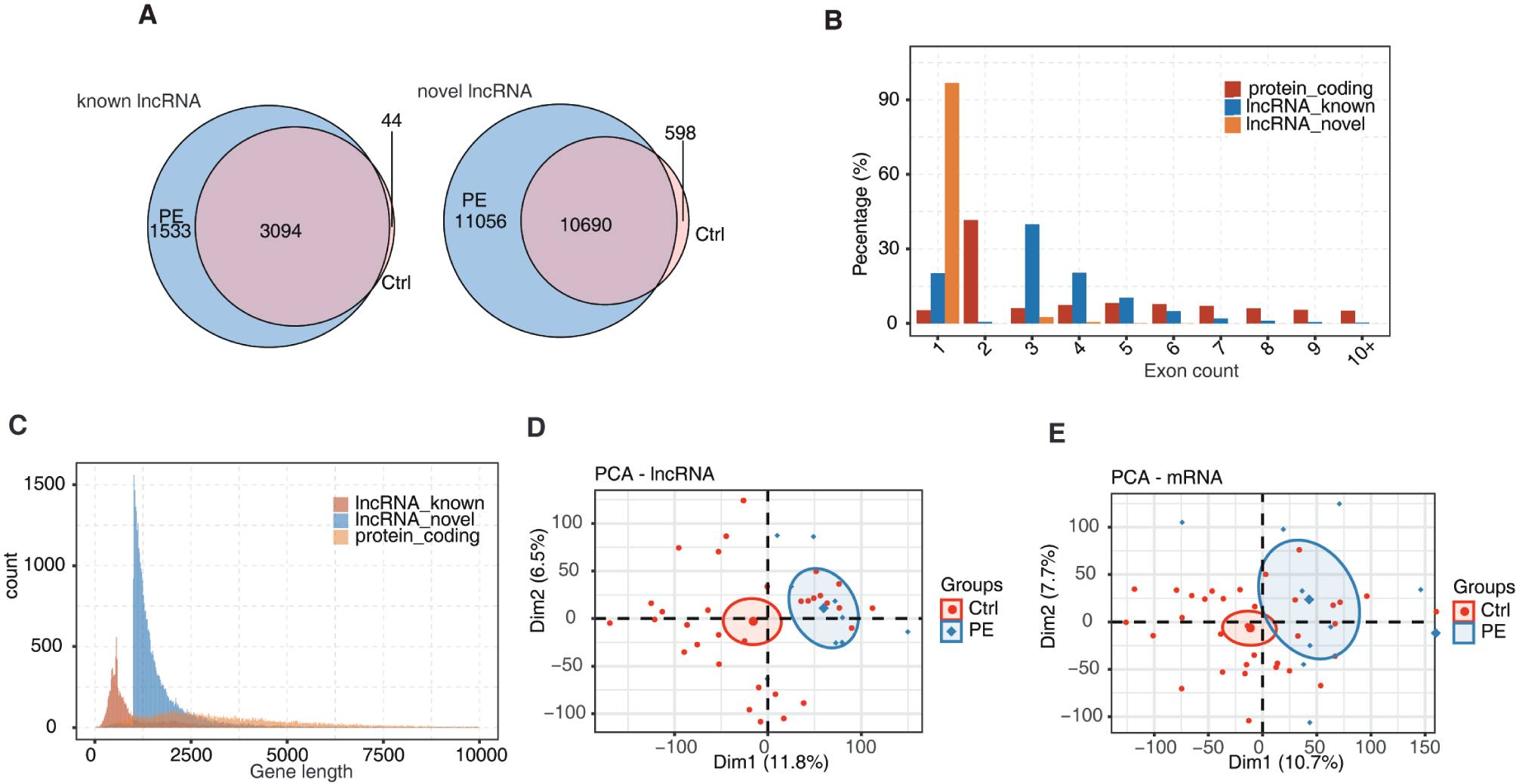
**

**Figure S1. Characteristics of lncRNAs idenfied in all samples from the GSE14821 dataset.** A. Venn diagram of known lncRNAs (left) and novel lncRNAs (right) with RPKM ≥ 0.2 in at least 2 samples of all GSE14821 samples. B. Distribution of exon count of known lncRNAs, novel lncRNAs, and protein-coding RNAs. C. Density of the length distribution of known lncRNAs, novel lncRNAs, and protein-coding RNAs. The length density distribution was generated by density function in R. (D, E). PCA of EPE and control placenta samples based on normalized lncRNA (D) and mRNA (E) expression. The samples were grouped by disease state. The ellipse indicates the 95% confidence regions of each group.


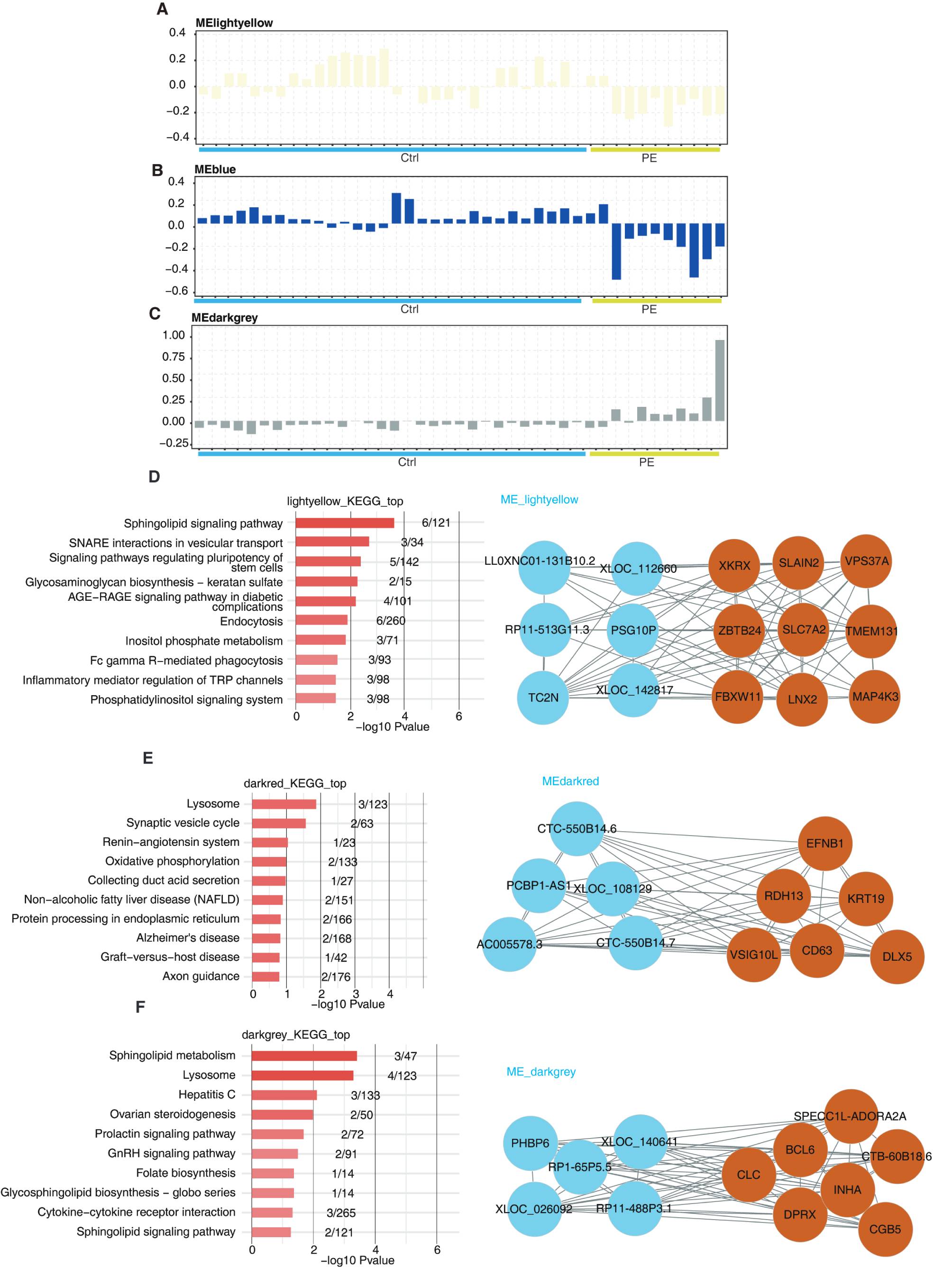


**Figure S2. WGCNA and KEGG analysis.** A–C. Eigengene bar graphs of yellow, blue, and dark gray modules. D–F. The network of hub mRNAs and lncRNAs (right) and the KEGG pathways enrichment assay (left) of yellow (D), dark red (E), and dark grey (F) modules. Brown circles indicate hub mRNAs. Blue circles indicate hub lncRNAs.
